# D2D Mobile Relaying Meets NOMA—Part II: A Reinforcement Learning Perspective

**DOI:** 10.3390/s21051755

**Published:** 2021-03-04

**Authors:** Safaa Driouech, Essaid Sabir, Mounir Ghogho, El-Mehdi Amhoud

**Affiliations:** 1 NEST Research Group, LRI Lab., ENSEM, Hassan II University of Casablanca, Casablanca 20000, Morocco; driouech.safaa@hotmail.fr; 2 Laoratoire de Reacherche en Informatique, Sorbonne Université, CNRS, LIP6, F-75005 Paris, France; 3 Department of Computer Science, University of Quebec at Montreal, Montreal, QC H2L 2C4, Canada; 4 TICLab, International University of Rabat, Rabat 11100, Morocco; mounir.ghogho@uir.ac.ma; 5 School of Computer Science, Mohammed VI Polytechnic University, Ben Guerir 43150, Morocco; elmehdi.amhoud@um6p.ma

**Keywords:** D2D relaying, 5G/B5G/6G, biform game, self-organized devices, Nash equilibrium, distributed reinforcement learning, NOMA/OMA

## Abstract

Structureless communications such as Device-to-Device (D2D) relaying are undeniably of paramount importance to improving the performance of today’s mobile networks. Such a communication paradigm requires a certain level of intelligence at the device level, thereby allowing it to interact with the environment and make proper decisions. However, decentralizing decision-making may induce paradoxical outcomes, resulting in a drop in performance, which sustains the design of self-organizing yet efficient systems. We propose that each device decides either to directly connect to the eNodeB or get access via another device through a D2D link. In the first part of this article, we describe a biform game framework to analyze the proposed self-organized system’s performance, under pure and mixed strategies. We use two reinforcement learning (RL) algorithms, enabling devices to self-organize and learn their pure/mixed equilibrium strategies in a fully distributed fashion. Decentralized RL algorithms are shown to play an important role in allowing devices to be self-organized and reach satisfactory performance with incomplete information or even under uncertainties. We point out through a simulation the importance of D2D relaying and assess how our learning schemes perform under slow/fast channel fading.

## 1. Introduction

### 1.1. Motivations and New Trends

The fifth-generation (5G) of mobile networks is being massively deployed worldwide, promising a never-before-seen user experience. 5G is expected to provide the basis for intelligent networks with some artificial intelligence (AI) applications and capabilities. However, a zero-touch network with fully intelligent orchestration and management remains elusive and will only be achieved in future [1]. In order to overcome the limitations of 5G deployment and fulfill the stringent requirements of future applications and services, the research community has started to explore and shape the next generation of wireless communications under different labels, such as B5G, 5GC, and 6G. In this context, some articles discussing the vision and open challenges for 6G have recently appeared in the literature, for example, refs. [2,3,4]. Indeed, 6G is foreseen to support novel data-hungry applications, a plethora of autonomous services and new communication scenarios by around 2030. These technologies will encompass holographic videos, extended reality (XR), flying networks and vehicles, massive unmanned mobility in three dimensions, autonomous driving, telemedicine, haptics, tactile Internet, brain-computing, large intelligent surfaces, connected autonomous systems, high-definition video streaming and cell-free communications, to name a few. Thus, the amount of wireless data traffic and the number of connected objects are expected to increase hundreds of fold per cubic-meter volume [5]. 6G will connect millions of users and billions of machines everywhere. Thus, many extreme requirements (low energy consumption, long battery life, high intelligence level, high speeds, far larger bandwidth than 5G, high reliability, low latency, high data rates, etc. [2,4,6,7]) need to be addressed by design.

It is a reality that the current centralized systems suffer from large overheads and heavy signaling that might greedily misuse the network’s scarce resources. Thus, a distributed system is recommended for dense and ultra-dense environments, as it offloads traffic from the network and minimizes the dependency on its connectivity. Decentralized decision-making exhibits important scalability features and can efficiently handle server breakdowns due to denial of service. One of the most important requirements and trends of the next generation of mobile networks is the self-X paradigm. This promises future networks will be able to perform the required functionalities, such as self-learning, self-configuration, self-optimization, self-healing, self-organization, self-aggregation, and self-protection, with no human intervention [8]. Such a paradigm shift is likely to lead to a more flexible and robust network. In this regard, artificial intelligence (AI) and machine learning (ML) can play significant roles in ensuring a fully connected digital society, supporting 6G autonomy, capturing insights and gaining proper comprehension of the environment. Namely, AI/ML will bring human-like intelligence into every aspect of networking operations (e.g., resource management; network planning and optimization; and failure detection and analysis). AI can be deployed and trained at the device and various network levels (base stations, core network, cloud, etc.). Of course, reaching this goal relies on the availability of data timely streamed to/from devices, in order to enhance operational reliability, real-time predictions and security.

Artificial intelligence has taken us by storm, helping us to make decisions in almost every sector and area of life. On the one hand, the most recent mobile handsets are already offering many exciting AI-based services, such as AI-based cameras, AI-based earphones and intelligent assistants (Siri, Cortana, Google Now, Bixby, Alexa, etc.) [8,9]. On the other hand, current assessment approaches closely rely on mathematical models that capture the structures and dynamics of the communication systems. However, efficient mathematical frameworks often do not offer high accuracy or use heavy mathematical tools with unsuitable computational time/complexity. Additionally, such an analytic model does not exist for some of the building blocks of wireless networks. Therefore, AI/ML will play a crucial role in 6G wireless networks, as it is capable of handling systems that cannot be modeled by standard mathematical frameworks. Now, ML-empowered machines can learn, adapt and improve their operations by exploiting the operational knowledge and experience gained in the form of data or experience. Based on the nature of available data and the explicitness of the learning objectives, an ML scheme can use supervised, unsupervised or reinforcement learning (RL).

Today, we live in a hyper-connected world where huge amounts of data are generated and can be efficiently used to fine-tune every single device’s decisions. Decentralized RL is required. By using that scheme, updating information on devices only requires local actions and observed payoffs. Such an algorithm is very important in stochastic/dynamic environments where many parameters are unavailable, unobservable or simply unknown. Centralized ML algorithms require a massive number of geographically distributed smart mobile devices to transmit their collected data to the cloud for training purposes. Due to privacy issues and communication evidence, it is impractical for all wireless mobile devices to transmit their local data to train the ML model. To cope with this and avoid costly training, it is important to introduce distributed, decentralized and edge-deployed learning ML algorithms, such as federated learning (FL) [10,11] and decentralized RL algorithms, that enable mobile devices to collaboratively learn a shared ML model without any explicit data exchange.

### 1.2. Our Contributions

In this article, we revisit the potential of game theory combined with machine learning to efficiently intelligentize network entities. More precisely, we devote the first part of this article [12] to predicting and analyzing the performance of a wireless network with self-organizing devices, using a biform game perspective. Of course, the system’s designer needs to have some information about the network, such as the game’s rules, number of users, available strategies, utility functions, game sequence, and external perturbing factors (random channel, mobility, etc.). In this paper, we implement two fully distributed reinforcement learning algorithms at the device level in order to reach stable equilibria.

We invite the reader to see part I [12] of this work for details on the game-theoretic aspects of our D2D relaying scheme. The main contributions of this work are fivefold:

**Part I’s**  contributions are related to performance analysis of a self-organizing D2D relaying scheme:
  1. We consider a hybrid two-tier scheme where cellular links use Non-Orthogonal Multiple Access (NOMA), whilst D2D links use Orthogonal Multiple Access (OMA). This scheme is suitable for both inband and outband D2D schemes.
  2. We fully characterize the Rayleigh channel model and derive closed forms for the outage probabilities of both OMA and NOMA links, and then compute the average throughput perceived by each device in the network.
  3. To the best of our knowledge, this work is the first to propose a biform game to capture the devices’ behaviors while deciding which radio access network (RAN) to connect (i.e., either cellular or D2D).
**Part II’s**  contributions are related to implementing self-organized mode selection using RL. Each device has to strategically learn when to select cellular mode and then act as a relay, and when it has to get access via D2D mode:
  4. We propose to empower devices with a self-organization capability, allowing them to reach pure Nash equilibria (linear reward-inaction) and mixed Nash equilibria (Boltzmann–Gibbs dynamics), in a fully distributed manner;
  5. We performed extensive simulations to analyze the effects of different parameters on the learning schemes. Insights on accuracy and convergence are also provided.

This article is organized as follows: A biform game and equilibrium analysis are provided in part I [12] of this research. Section 2 presents a comprehensive literature review. The problem is formulated in Section 3. The decentralized RL algorithms are presented in Section 4. Next, we discuss the scalability, convergence and insights of our algorithms in Section 5. Extensive learning simulations are provided in Section 6. Finally, we draw some concluding remarks and list future directions in Section 7.

## 2. Related Work

Reinforcement learning (RL) is built upon a feedback reward mechanism: At every time step of the learning process, the agent interacts with the environment and observes its state, takes action from the set of available actions that maximizes the long-term cumulative reward and eventually moves to the next state. Some of the well-known techniques developed for network management are the multi-arm bandit, temporal-difference learning, the Markov decision process, SARSA learning, Q-learning and Monte Carlo methods [13,14,15]. These techniques can be applied in the application layer for proactive caching, data offloading, error prediction, data rate allocation and security. In the network layer, RL can be used for multi-objective routing, packet scheduling, traffic prediction and classification, etc. In the physical layer, RL can be employed for link preservation, channel tracking, radio access association, on-demand beamforming, energy harvesting, modulation selection, radio identification, etc. [8,13,16,17,18].

RL consists of a pre-programmed process run by an agent seeking to maximize its cumulative reward over time. It has received substantial attention and has been applied to wireless networks at numerous levels [13,19,20]. The authors of [18] came up with a distributed RL algorithm that dynamically changes the transmitted data and power control at each sensor node according to observed state information such that the data of all sensor nodes are received while minimizing the delay. The authors in [21] came up with a distributed RL-based resource allocation scheme to mitigate interference between D2D users and cellular users. A distributed spectrum allocation framework based on multi-agent deep reinforcement learning was also proposed in [22]. Additionally, the authors in [23] proposed learning schemes that enable cognitive users to jointly learn their optimal payoffs and strategies for both continuous and discrete actions. The authors in [24] proposed an actor–critic reinforcement learning scheme for downlink transmission based on radio resource scheduling policy for long term evolution—advanced (LTE-A), to accomplish resource scheduling efficiently by maintaining user fairness and high QoS capabilities. In [25], the authors proposed a reinforcement learning scheme to optimize routing strategy without human participation. In [26], an RL algorithm was used for distributed packet routing. The latter resulted in less congestion and shorter delivery times.

Most of the related literature on reinforcement learning algorithms focuses on optimizing network performance. We aimed to study and analyze the system to predict the network’s outcome from a biform game-theoretic perspective. Then, we used decentralized learning schemes to learn the options each device should pursue to earn the “best” long-term average profit. On the one hand, game theory allows capturing the behavior of devices and finding stable solutions assuming complete information. On the other hand, RL schemes are used to reach these solutions, only using local information and some observable, possibly perturbed/random metrics.

## 3. The System

**Figure 1:**
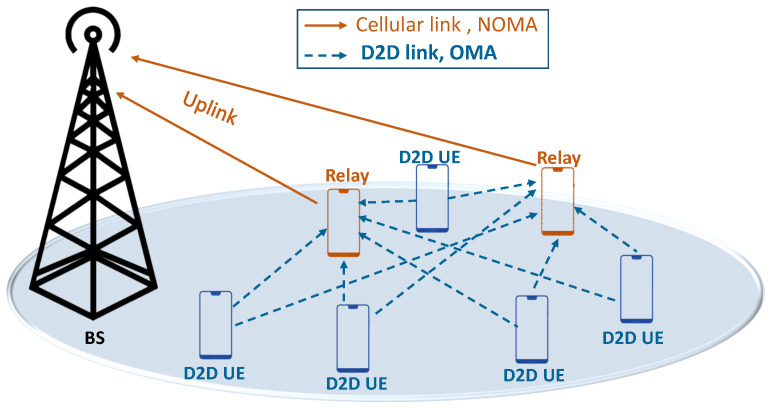
Cellular offloading using device-to-device (D2D) cooperative relaying.

Consider the uplink case of a single 4G/5G/6G cell, where a finite number of devices N={1,2,....,n} are randomly distributed around the serving Base Station (BS). The devices communicate using NOMA in cellular links combined with conventional OMA for D2D links, as shown in Figure 1. We use a separate band for D2D users (i.e., D2D overlay mode). We use OMA for D2D links to (1) study a hybrid access system, and (2) eliminate interference between cellular and D2D UE. Each device *i* transmits using power vector $P_{i}=\left(P_{i}^{c},P_{i}^{d}\right)$. In this article, we do not consider multi-homing. When the mobile *i* is connected to the BS, it transmits with power $P_{i}^{c}$, which implies $P_{i}^{d}=0$. The mobile transmits with power $P_{i}^{d}$ (i.e., $P_{i}^{c}=0$) if it is connected through a D2D link. Of course, the transmit power using a D2D link is (much) lower than in cellular communication. Each device is $d_{i}$ away from the BS and experiences a channel gain $h_{i}$. For better readability, the main notation and symbols used in this article are listed in Table 1.

For the sake of simplicity and without loss of generality, the device numbered 1 is assumed to be the closest device to the serving BS, with distance $d_{1}$. It transmits with the lowest power $P_{1}$ and experiences the strongest channel gain $h_{1}$. It follows that device *n* is the farthest with distance $d_{n}$ from the BS; it uses the highest transmit power $P_{n}$ and uses the weakest channel $h_{n}$. Namely, we have $|h_{1}{|}^{2}\geq |h_{2}{|}^{2}\geq \cdots \geq |h_{n-1}{|}^{2}\geq {\left|h_{n}\right|}^{2}$. Let w(t) be the received noise at the BS and assume each device *i* transmits its individual signal $s_{i}\left(t\right)$. Then, the aggregate received signal at the BS is:

(1) $$S\left(t\right)=\sum_{i=1}^{n}\sqrt{P_{i}^{c}}h_{i}s_{i}\left(t\right)+w\left(t\right),$$

The BS decodes the signals by applying the successive interference cancellation (SIC) technique. The received signal power corresponding to the user of the strongest channel is likely the strongest at the BS, so the signal is the first to be decoded at the BS and experiences interference from all the remaining weaker channels in the cluster [27,28]. Thus, the transmission of device 1 experiences interference from users with weaker channels in the cluster, whereas the transmission of device *n* experiences zero interference. In contrast to downlink NOMA, each user in uplink NOMA can independently utilize its battery power to the maximum since the channel gains of all the users are sufficiently distinct [29].

### 3.1. Channel Model

In this article, the radio signal experiences attenuation due to the path-loss with exponent α and Rayleigh fading. We denote by $\gamma_{i}$ the instantaneous signal to interference and noise ratio (SINR) of device *i*, and it is given by:

(2) $$\gamma_{i}=\frac{P_{i}^{c}{\left|h_{i}\right|}^{2}d_{i}^{-\alpha_{c}}}{\sum_{j=i+1}^{n}P_{j}^{c}{\left|h_{j}\right|}^{2}d_{j}^{-\alpha_{c}}+\sigma_{N}^{2}},$$

It is worth noting that the SINR of the weakest device *n* experiences no interference according to NOMA operation; i.e., $\gamma_{n}=\frac{P_{n}^{c}{\left|h_{n}\right|}^{2}d_{n}^{-\alpha_{c}}}{\sigma_{N}^{2}}$. $\sigma_{N}^{2}$ denotes the variance of the thermal additive white Gaussian noise. When devices communicate through a D2D link, the SINR of a device *j* relaying on device *i* is given by:

(3) $$\gamma_{j}=\frac{P_{j}^{d}{\left|h_{j}^{d}\right|}^{2}d_{j,i}^{-\alpha_{d}}}{\sum_{k\in N\backslash \{i,j\}}^{n}f_{j,k}P_{k}^{d}{\left|h_{k}^{d}\right|}^{2}d_{k,i}^{-\alpha_{d}}+\sigma_{N}^{2}},$$

where $f_{j,k}$ is the orthogonality factor between the carriers allocated to active devices *j* and *k*. *k* refers to the other devices communicating through the D2D link and relaying on device *i*. Each device aims at guaranteeing an instantaneous SINR above a certain threshold $\gamma_{i,th}$ to have successful communication. The outage probability denotes the probability that the SINR is less or equal than a given SINR threshold ($\gamma_{i,th}$). The outage probability $P_{i}^{out}\left(\gamma_{i}\right)$ of device *i* is given by [12]:

(4) $$P_{i}^{out}\left(\gamma_{i}\right)=Pr\left(\gamma_{i}\leq \gamma_{i,th}\right)=1-\frac{\prod_{j=i+1}^{n}\lambda_{j}.e^{-\frac{\gamma_{i,th}\sigma_{N}^{2}\lambda_{i}}{P_{i}^{c}d_{i}^{-\alpha_{c}}}}}{\prod_{j=i+1}^{n}\left(\lambda_{j}+\frac{\gamma_{i,th}P_{j}^{c}d_{j}^{-\alpha_{c}}}{P_{i}^{c}d_{i}^{-\alpha_{c}}}\lambda_{i}\right)},$$

where $\frac{1}{\lambda }$≥ 0 is the mean and scale parameter of the distribution, often taken as equal to 1. The calculus of the outage probability in cellular and D2D modes was detailed in the first part [12] of this work.

### 3.2. Average Throughput

In general, device *i* transmits data with a rate $R_{i}$ with every use of the channel (i.e., in every packet or frame transmission), with the condition that $R_{i}$ must not exceed its channel capacity; i.e., $R_{i}\leq \log\left(1+\gamma_{i}\right)$.

We define the throughput of the transmission as the rate of successful data bits that are transmitted to the destination over a communication channel, expressed as follows:

(5) $$\Theta_{i}\left(\gamma_{i}\right)=\frac{M}{L}R_{i}\left(1-P_{i}^{out}\left(\gamma_{i}\right)\right)=\rho_{i}\left(1-P_{i}^{out}\left(\gamma_{i}\right)\right),$$

where $\rho_{i}=\frac{M}{L}R_{i}$. *M* is the data length (in bits). *L* denotes the total number of bits in a frame.

## 4. Decentralized Learning Algorithms

In this section, we propose an algorithmic implementation of the biform game framework we developed in [12], by means of decentralized learning algorithms. Each device that is a player in the game operates in a self-organized manner using a decentralized RL algorithm to learn its locally optimal (at Nash equilibrium) strategy over time. Based on that, it converges to the action (communication mode) that improves its throughput. The optimal action is defined as the one with the highest expected environmental reaction value. Decentralized RL learning algorithms are designed to naturally converge to Nash equilibria in games with incomplete information.

**Figure 2:**
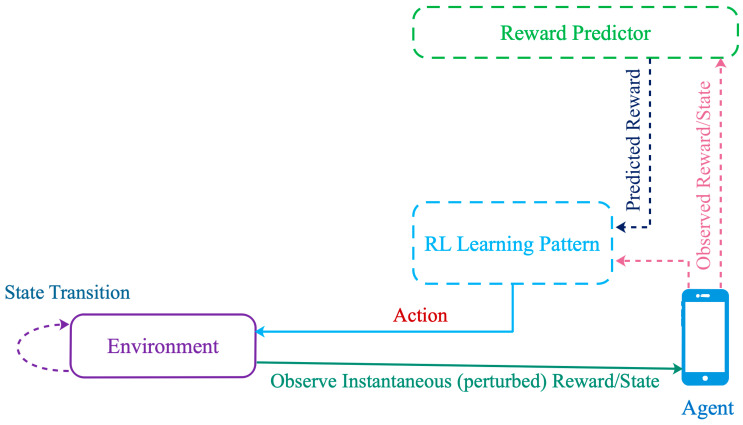
A decentralized reinforcement learning process.

A learning scheme is a process within which an agent interacts with the environment and updates its strategy toward a Nash equilibrium, based on its experience built from past rounds. Each device can learn the optimal action from a finite set of actions by iterating its update pattern, even under bounded uncertainty; see Figure 2. Such a decentralized scheme only requires local information and some metrics observed while interacting with the environment [15].

Let us consider *n* agents representing the *n* active devices in the network, where $A^{t}$= $\left\{a_{1}^{t},a_{2}^{t},\cdots ,a_{n}^{t}\right\}$ is the set of all joint agent actions at time *t*. At the beginning of every time slot/step, each device *i*, i∈N, chooses either to be a relay ($a_{i}^{t}=0$) or to communicate through D2D ($a_{i}^{t}=1$) while expecting other devices will serve as relays. $U_{i}^{t}$ is the average throughput observed/measured by device *i* at the end of time slot *t*; it represents its payoff (reward) at time *t*. Let $p_{i}\left(t\right)$ denote the probability distribution of the i−th agent over its pure strategies at instant *t*; $p_{i}\left(0\right)$ is the initial mixed strategy of player *i*. Each round of the game consists of each of the players choosing an action randomly and independently based on their respective current strategies and their perceived rewards at the previous step.

The update process of the distributed learning algorithm is depicted in Figure 2. Each device/player constructs and updates its strategy based on its set of own-actions, received reward (i.e., observed/measured throughput) and potentially some belief or estimate of future reward. There is no explicit communication or information exchange between active devices. Here, all the players update their strategies within the same time window in a dynamic interactive fashion. The wireless environment is perturbed by the channel state, devices’ mobility patterns, devices’ locations, interference, devices’ actions, etc.

### 4.1. Learning Pure Nash Equilibrium (PNE): The Linear Reward-Inaction Algorithm (LRI)

In order to reach a pure equilibrium in a distributed manner under unknown and slow fading channel assumptions, with $|h_{1}{|}^{2}\geq |h_{2}{|}^{2}\geq \ldots .\geq |h_{n-1}{|}^{2}\geq {\left|h_{n}\right|}^{2}$, we propose to use the linear reward-inaction (LRI) algorithm for the devices to learn their respective best response strategies. Recall that LRI is a completely decentralized learning algorithm, since each learner ignores information from other agents and only considers its own actions and payoffs from the environment. Every device, independently of its competitors, updates a probability distribution over time to gradually converge to the best strategy. Initially, the player chooses randomly a strategy according to the initial probability distribution; after each time step, the algorithm increases the probability of picking strategies with great utility and decreases (reinforcement) the probability of choosing the other strategies. Let **p**${}_{t}=\left(p_{t},1-p_{t}\right)$ be the probability distribution at instant *t*, where $p_{t}$ denotes the probability of choosing the action “relay” at instant t; thus, $1-p_{t}$ is the probability of choosing the action “D2D”.

After each transmission, the device receives an acknowledgment (ACK) if the BS successfully receives the message sent in that slot. Otherwise, the receiver feeds back an explicit/implicit NACK. Thus, the main question is: what is the action that can allow a device to receive the maximum possible number of ACKs with every use of channel?

To receive an ACK from the BS, the SINR $\gamma_{i}\left(t\right)$ experienced at time *t* of device *i* needs to be higher than the SINR threshold $\gamma_{th}$. To model this, we define the reward of agent *i* for a given action profile $\left(a_{i}\left(t\right),a_{-i}\left(t\right)\right)$ at time slot *t* as:

(6) $$\begin{array}{cc} U_{i}^{t}\left(a_{i}\left(t\right)\right)= & Thp_{i}\left(t\right)*{𝟙}_{\left\{\gamma_{i}\left(t\right)\geq \gamma_{th}\right\}}=\left\{\begin{array}{c} Thp_{i}\left(t\right)\;if\;\gamma_{i}\left(t\right)\geq \gamma_{th}\;\left(\mathrm{ACK}\right)\;\\ 0\;\;\;else\;\;\;\left(\mathrm{NACK}\right) \end{array}\right. \end{array}$$

where $Thp_{i}\left(t\right)$ is the instantaneous throughput perceived by device *i* at time instance *t*, and ${𝟙}_{\cdot }$ is the indicator function. Of course, the instantaneous throughput depends on the device’s strategy, other devices’ strategy vectors, environment perturbations, etc. M denotes the set of devices directly connected to the base station at instant *t*, with cardinality m(t). Thus, the remaining n−m(t) devices are getting access via D2D.

(7) $$Thp_{i}\left(t\right)=\left\{\begin{array}{cc} \rho_{i}, & \mathrm{if}\;\mathrm{all}\;\mathrm{the}\;\mathrm{devices}\;\mathrm{choose}\;\mathrm{cellular},\\ x_{i}\rho_{i}, & \mathrm{if}\;\mathrm{device}\;i\;\mathrm{is}\;a\;\mathrm{relay},\\ \sum_{j\in M}^{}\frac{1-x_{j}}{n-m(t)}\rho_{j}, & \mathrm{if}\;\mathrm{device}\;i\;\mathrm{get}\;\mathrm{access}\;\mathrm{through}\;D2D\;\mathrm{links},\\ 0, & \mathrm{No}\;\mathrm{device}\;\mathrm{is}\;\mathrm{connected}\;\mathrm{to}\;\mathrm{the}\;\mathrm{base}\;\mathrm{station}. \end{array}\right.$$

In case there are many devices communicating through D2D mode, the fraction of throughput given from the relays will be equally divided between the D2D-transmitters. If there is at least one device in the D2D group, then the relay device allocates a fraction of its throughput $1-x_{i}$ to that group. $x_{i}$ allows one to define the mode selection and the cooperation level of device *i*. For instance, $x_{i}=1$ means device *i* fully opts for cellular mode. Meanwhile, $x_{i}=0$ means device *i* chooses to communicate through D2D link. When $x_{i}\in ]0,\;1[$ the device *i* plays the role of a relay. If $x_{i}$ is high then device *i* uses a high fraction for itself and does not relay much; otherwise, it offers more throughput to D2D devices.

According to LRI pattern, the probability of device *i* serving as a relay at instant t+1 is:

(8) $$p_{i}\left(t+1\right)=p_{i}\left(t\right)+\epsilon_{t}\frac{U_{i}^{t}\left(a_{i}\left(t\right)\right)}{R}\left({𝟙}_{\left\{i\;\mathrm{is}\;a\;\mathrm{relay}\;\mathrm{at}\;\mathrm{instant}\;t\right\}}-p_{i}\left(t\right)\right),i\in N.$$

- $p_{i}\left(t\right)$ is the probability of relaying for device *i* at time t.
- $\epsilon_{t}$ denotes the learning rate parameter at instant t (i.e., the step size).
- $\frac{U_{i}^{t}}{R}$ is the environmental response, which is represented here as the fraction between the device *i*’s instantaneous throughput and the transmission rate.
- ${𝟙}_{t}$ denotes the indicator function that equals 1 when the action chosen by a device at time *t* is $a_{i}=0$; otherwise it equals 0.

The learning algorithm described in Algorithm 1 used by each of the players is explained below:

1. At every instant, each player chooses an action based on its action probability vector. Thus, player *i* chooses action $a_{i}$ at instant *t*, according to the probability distribution **p**${}_{i}\left(t\right)$;
2. Each player obtains a reward based on its action and the set of all other devices’ actions. The reward of player *i* is $U_{i}^{t}\left(a_{i}\left(t\right)\right)$;
3. Each player updates his action probability based on the rule in Equation (8).

**Algorithm 1:** Linear reward-inaction algorithm (LRI).      **Parameter Initialization**      Each device peaks randomly an initial probability of relaying $p_{i}^{0}$.      **In parallel:** at each device *i* **do**      **Repeat**            **if** $a_{i}\left(t\right)=0$ **then**                     $p_{i}^{t+1}\leftarrow p_{i}^{t}+\epsilon \frac{U_{i}^{t}}{R}\left(1-p_{i}^{t}\right)$            **else**                     $p_{i}^{t+1}\leftarrow p_{i}^{t}-\epsilon \frac{U_{i}^{t}}{R}p_{i}^{t}$      **Until** The stopping criterion

### 4.2. Learning Mixed Nash Equilibrium (MNE): Boltzmann–Gibbs-Based Payoff-RL

Under the channel randomness assumption (i.e., fast fading channel), each device decides to be indifferent between the actions chosen, and look for the probability of relaying that guarantees the same payoff of either being connected to cellular or D2D. After that every device has chosen an action according to its probabilities, it receives the corresponding payoff and then updates its strategy. The update of strategies occurs following Boltzmann–Gibbs-based Payoff-RL [13]. The players use only information regarding their own choices and perceived payoffs, and do not explicitly require external information concerning the channel gains, the transmit powers, the payoffs, and choices of the other players, i.e., a fully distributed learning algorithm.

The Boltzmann–Gibbs-based Payoff-RL algorithm consists of updating the probability distribution over the possible actions as follows:

(9) $$\left\{\begin{array}{c} p_{i}\left(t+1\right)=\frac{e^{\frac{{\hat{U}}_{i}^{t}\left(a_{i}\right)}{r_{i}}}}{\sum_{a_{i}^{{}^{\prime }}\in A_{i}}e^{\frac{{\hat{U}}_{i}^{t}\left(a_{i}^{{}^{\prime }}\right)}{r_{i}}}},a_{i}\in A_{i},i\in N\;\\ {\hat{U}}_{i}^{t+1}\left(a_{i}\right)={\hat{U}}_{i}^{t}\left(a_{i}\right)+\nu_{i,t}{𝟙}_{\left\{a_{i,t}=a_{i}\right\}}\left(U_{i}^{t}\left(a_{i}\right)-{\hat{U}}_{i}^{t}\left(a_{i}\right)\right) \end{array}\right.$$

where $r_{i}$ is player *i*’s temperature. This quantity measures the rationality level of *i*. Under small values of $r_{i}$, the player is strongly rational and the algorithm converges to the Nash equilibrium. Whereas, the player becomes irrational under large values of $r_{i}$, which results in selecting available strategies following a uniform distribution. Thus, the algorithm fails to explore the equilibrium strategies. $\nu_{i,t}$ denotes the learning rate: it counts how many times the actions $a_{i}$ have been chosen.

The Boltzmann–Gibbs dynamics are summarized in Algorithm 2. In addition to the observed real reward, this learning scheme allows the players to learn mixed strategies by estimating/predicting the payoffs at next time steps. The reward predictor is adjusted using actual real reward, which improves the estimation accuracy.
**Algorithm 2:** Boltzmann–Gibbs-based payoff-RL.      **Parameter Initialization**      Each device peaks randomly an initial probability of relaying $p_{i}^{0}$.      **In parallel:** at each device *i* **do**      **Repeat**                  $p_{i}^{t+1}\leftarrow \frac{e^{\frac{{\hat{U}}_{i}^{t}\left(a_{i}\right)}{r_{i}}}}{e^{\frac{{\hat{U}}_{i}^{t}\left(a_{i}\right)}{r_{i}}}+e^{\frac{{\hat{U}}_{i}^{t}\left({\bar{a}}_{i}\right)}{r_{i}}}}$                  ${\hat{U}}_{i}^{t+1}\left(a_{i}\right)\leftarrow {\hat{U}}_{i}^{t}\left(a_{i}\right)+\nu_{i,t}{𝟙}_{\left\{a_{i,t}=a_{i}\right\}}\left(U_{i}^{t}\left(a_{i}\right)-{\hat{U}}_{i}^{t}\left(a_{i}\right)\right)$      **Until** The stopping criterion

## 5. Some Insights on RL Convergence, Fairness and Scalability

While building an iterative scheme, the system designer must deal with many important issues in order to yield an efficient learning algorithm. The efficiency of such a scheme is measured through its accuracy, speed, fairness and scalability features.

### 5.1. Convergence

The learning algorithm converges when as the iterations proceed, the output gets closer to some specific stationary value. After that, the algorithm settles down on this value and does not improve anymore even when additional training is applied. From a theoretic perspective, most learning algorithms found in the literature fail to converge to the value computed analytically. Indeed, these iterative schemes provide an output that always deviates from theoretic values by some amount. However, in numerous cases a near-optimal solution is acceptable. Here, relative error can be a suitable stopping criterion, i.e., when $\left|\frac{p_{i}^{t+1}-p_{i}^{t}}{p_{i}^{t}}\right|\leq \epsilon$, where ϵ is the convergence tolerance that sets the desired accuracy level. It is understood that high accuracy might result in a long convergence time. In some specific cases, the RL algorithm might not converge or simply can diverge: (1) when the speed of convergence is arbitrary slow; or (2) when no attractor point (e.g., Nash equilibrium) exists. In [13], the convergence issue has been extensively discussed. In general, many parameters can affect the speed and accuracy of convergence of the learning algorithms, namely, the number of players, the mobility, the randomness degree of the environment, etc. In this work, we present some parameters that impact the time of convergence of the proposed algorithms, namely, fast/slow fading, power allocation, SINR threshold and the learning rate parameter.

### 5.2. Fairness

In this article, we fully characterize the pure and mixed Nash equilibria. Nash Equilibrium is defined as a solution where no player can improve its reward by deviating unilaterally, given the actions of the other players. At the PNE, each device must select to exclusively connect to the serving eNodeB or to exclusively connect via D2D. Such a solution presents evident fairness issues. In fact, devices communicating through D2D may transmit at low power due to short links. Meanwhile, the relaying devices consume much higher power, as they have to reach the eNodeB from longer distances and are expected to share, via D2D links, their throughput with unconnected devices. Of course, lack of fairness may induce harmful stability issues and then may result in a breakdown of the whole system (e.g., no device accepts to serve as a relay). Fortunately, mixed Nash equilibrium exhibits better fairness properties, as it allows each device to freely use its available strategies with different probabilities to possibly ensure a stable situation and satisfactory performance.

### 5.3. Scalability

Scalable algorithms are designed to handle huge amounts of data and perform heavy computations in a cost-effective and fast manner. The tremendous quantity of data that is being generated by IoT, 5G and the future 6G, is openly challenging vendors, operators and wireless research community. Hence, scalability is becoming a prominent topic of focus to conduct exciting researches. Efficient proposals able to deal with massive number of devices and huge data within a reasonable time window are of paramount importance. It is clear that enabling decision-making at wireless devices is beneficial in terms of spectrum utilization, reduced signaling and computational/energy costs [13]. Our schemes are designed to be fully decentralized, so independently of the number of devices. Since the device only uses local information and some observed reward/state, then our RL schemes exhibit nice elasticity property. Our RL-based algorithms are expected to continue performing well at the cost of longer convergence time as the population size increases. Still, thorough scalability analysis has to be conducted to analyze how does our schemes scale up in ultra-dense environments. Prior to designing an RL scheme, one needs to first predict and analyze the agents behavior [12].

## 6. Performance Analysis

In this section, we evaluate the performances of the RL algorithms using Mathworks Matlab R2020a. For illustrative purposes, we performed simulations for the three-device case. Unless counter-indication, figures were produced using the following setup: $P_{1}^{c}=$10 mW, $P_{2}^{c}=$30 mW, $P_{3}^{c}=$50 mW, $P_{1}^{d}=P_{2}^{d}=P_{3}^{d}=$ 5 mW, $R_{1}=R_{2}=R_{3}$ = 1 Mbit/s, *L* = *M* = 1024 bits, $\gamma_{th}$ = 40 dB, $\alpha_{c}=\alpha_{d}=3$ and $\sigma_{N}^{2}=-116$ dBm, $d_{1}=100$ m, $d_{2}=300$ m, $d_{3}=500$ m, $f=10^{-5}$, $x_{1}=x_{2}=x_{3}=0.5$. As for the channel gains, we consider Rayleigh channels such that $E\left[h_{1}\right]\geq E\left[h_{2}\right]\geq E\left[h_{3}\right]$.

### 6.1. Reaching a Pure Nash Equilibrium Using the Linear Reward-Inaction Algorithm

**Figure 3:**
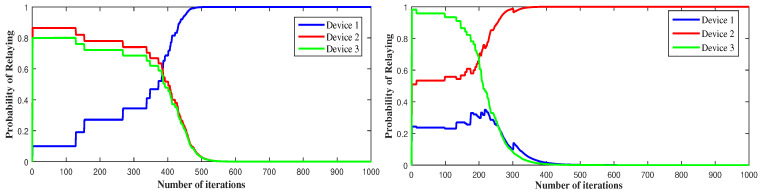
Convergence to pure Nash equilibrium using the linear reward-inaction algorithm.

Figure 3 depicts the use of LRI learning algorithm to reach the PNE. It is clearly seen that device 1 converges to $a_{1}^{*}=1$ while device 2 and 3 converge to $a_{2}^{*}=a_{3}^{*}=0$. So the action that verifies a PNE is (0,1,1), which means that device 1 serves as a relay while device 2 and device 3 connect to the D2D link. It also converges, sometimes, to the other pure equilibria where device 2 communicates through cellular and serves as relay to the other devices. We shall mention that our LRI-based scheme converges to the same pure equilibria found using the framework developed in part I [12]. It converges more frequently to the dominant strategy when exists, independently of the initial probabilities of learning.

**Figure 4:**
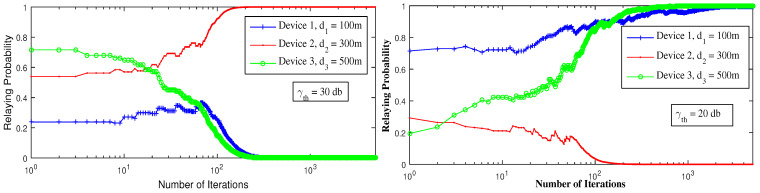
Convergence to pure Nash equilibrium using LRI, for different SINR threshold values ($\gamma_{th}$).

Figure 4 reports the change in the devices’ strategies with respect to the SINR threshold. To attain the equilibrium, device 2 decides to serve as a relay in the first figure when $\gamma_{th}$ decreases, while in the second figure device 1 and 3 serve both as relays and device 2 remains in D2D. Decreasing the SINR threshold increases the probability of the devices to have successful communication and to receive an ACK. For $\gamma_{th}=40$ dB, the equilibrium is reached when device 1 is the relay, because, as the strongest device, it can attain such threshold and can be a relay to device 2 and device 3. For $\gamma_{th}=30$ dB, device 2 can also have a successful communication if device 3 is in D2D, in this way device 1 gets affected by device 2’s interference and decides to switch to D2D for better throughput. For $\gamma_{th}=20$ dB, all the devices can have successful transmissions, but to reach the equilibrium, device 1 and device 3 serve as relays while device 2 is in D2D mode. In this way, device 2 gets rid of all interference and does not interfere with device 1 signal. Additionally, device 3 transmits with no interference.

**Figure 5:**
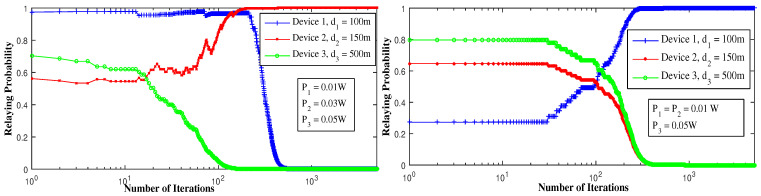
Convergence to pure Nash equilibrium using LRI, for different transmit powers of device 2.

Figure 5 shows the effect of the devices’ distances and the transmit power to the equilibrium strategies. In this case, device 2 becomes closer to the BS, so interference applied from device 2 to device 1 increases. This explains device 2 taking the relay role as it is the second strongest device. Additionally, the transmit power plays an important role in defining the equilibrium strategies. However, when $P_{2}$ decreases, interference effect decreases and allows device 1 to take over again the relay role.

**Figure 6:**
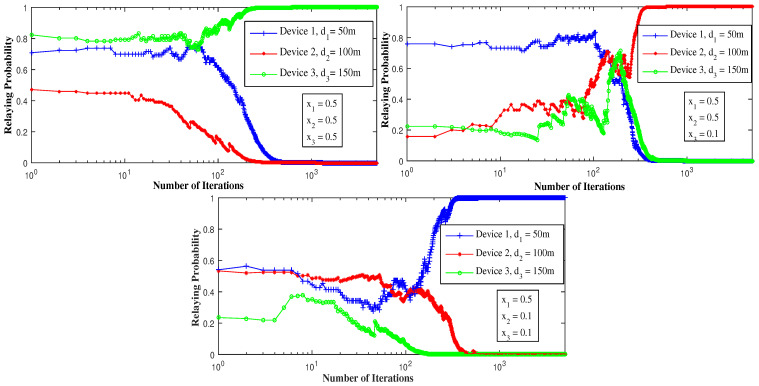
Convergence to pure Nash equilibrium using LRI, for different cooperation levels ($x_{i}$).

In Figure 6, we see that device 3 decides to serve as a relay when its distance from the BS decreases. By doing so, interference applied from device 3 to device 2 increases, which decreases $\gamma_{2}$ that is why device 3 serves as a relay. Note that device 3 is the weakest but at the same time it is the only device that doesn’t interfere with the other devices’ signals, once on the cellular side. It prefers, then, to serve as a relay than to be in D2D and get affected by other devices’ interference. However, minimizing the fraction of throughput device 3 gets for itself, if it serves as a relay, minimizes its throughput and ruins the equilibrium. That is why device 2 takes over the relaying mission in the second figure when $x_{3}$ decreases. For the same reason, device 1 serves as a relay when $x_{2}$ and $x_{3}$ decreases. This observation shows the importance and effectiveness of the decentralized RL algorithm to allow the devices to attain the equilibrium by converging to the pure action strategy.

**Figure 7:**
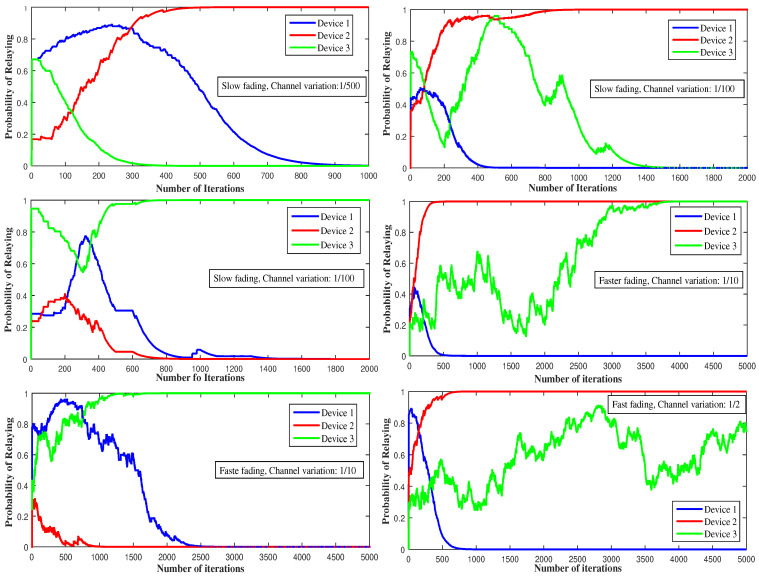
The effect of channel fading on the convergence toward a pure Nash equilibrium using LRI.

Figure 7 depicts the effect of the channel variation on the time of convergence of the learning algorithm. The higher the speed of variation of the channel, the longer the algorithm takes to converge. Additionally, due to the random aspect of the channel, the algorithm might not converge to the same equilibrium. In addition, for the fast-fading case, the algorithm does not converge at all; it continues to seek pure equilibrium but cannot reach it due to the rapid variation of the channel. Henceforth, we need to look at the mixed Nash equilibrium to suggest an alternative solution when a PNE may not exist, or the channel is rapidly varying, slowing-down or preventing convergence.

### 6.2. Reaching Mixed Equilibrium Using Boltzmann–Gibbs (BG) Algorithm

Different from a pure strategy that denotes the action a device takes with probability 1, a mixed strategy is an assignment of a probability to each pure strategy. In the pure analysis, the equilibrium is reached such that no player would benefit by changing his strategy, while in the mixed analysis each device is indifferent between the actions taken. In the sequel, we investigate the mixed strategy using BG-RL algorithm and the long-term behavior of the devices.

In part I [12] of this work, we rely on the expected throughput value (through outage probability) and the average channel gain. Now, we study the real case by generating random Rayleigh channel gains. We respect the condition of ${\left|h_{1}\right|}^{2}\geq {\left|h_{2}\right|}^{2}\geq {\left|h_{3}\right|}^{2}$ and allow random fast fading. To reach the mixed equilibrium, we use the decentralized Boltzmann–Gibbs dynamics above-mentioned in Algorithm 2.

**Figure 8:**
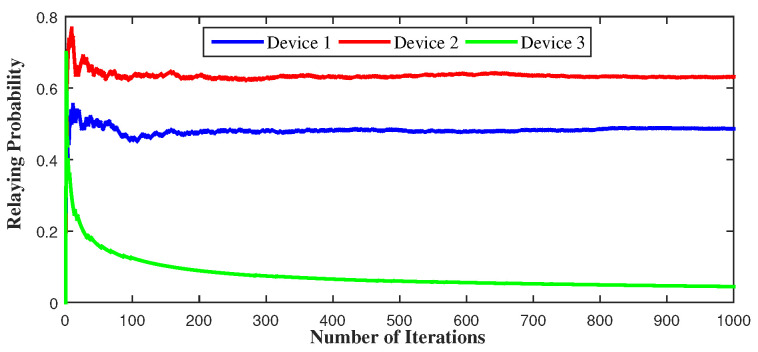
The effect of channel fading on the convergence to a mixed NE using BG dynamics.

Figure 8 provides an illustrative example of $p_{i}^{*}$ convergence, under Gibbs-Boltzmann scheme. It discovers the mixed equilibrium strategies in the case of a fast-fading channel. The proposed scheme, contrary to the biform game, considers random channels and estimated utilities. In the figure, device 2 serves as a relay for most of the time (65% of the time), then device 1 with 45% and finally device 3 with 5%. Device 1 is the strongest but it is the most affected by both devices’ interference. By adopting the indifferent mode, it chooses to communicate through cellular for half of the time and D2D for the other half. While device 2, the second strongest device, chooses to communicate through cellular and serve as a relay for most of the time. Device 2 is affected, once in cellular, by device 3 interference only. Thus, to reach high throughput, device 3 needs to stay in D2D mode, this is why device 3 communicates through D2D for more than 90% of the time.

**Figure 9:**
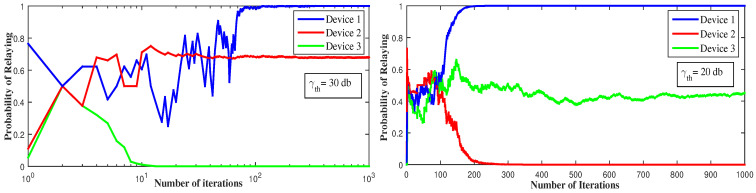
The effect of the SINR threshold ($\gamma_{th}$) on the convergence to a mixed NE using BG dynamics.

**Figure 10:**
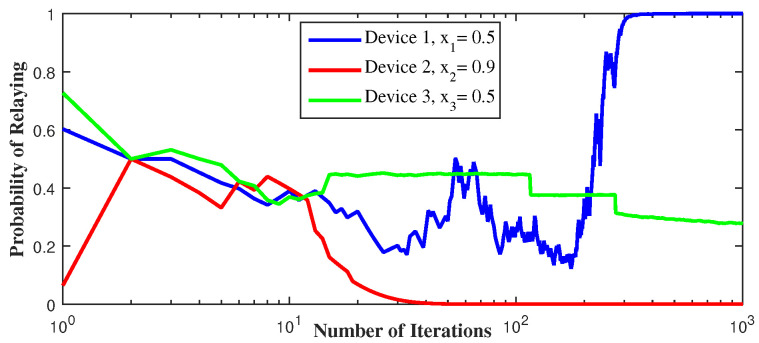
The effect of cooperation level ($x_{i}$) on the convergence to a mixed NE using BG dynamics.

Figure 9 and Figure 10 depict the behavior of the devices using the BG algorithm function of $\gamma_{th}$ and $x_{i}$. Differently from Figure 8 where $\gamma_{th}=40$ dB and $x_{i}=0.5$, i∈{1,2,3}, the algorithm converges to partially mixed equilibria. For $\gamma_{th}=30$ dB, device 1 decides to always communicate through cellular and serve as relay to device 3 that decides to always use D2D communication, while device 2 communicates 70% of the time via cellular mode and 30% via D2D. For $\gamma_{th}=20$ dB, device 1 behaves as before and serves as relay to device 2 that decides to always use D2D communication, while device 3 communicates through both modes. The reason behind this behavior is that device 1, as the strongest device, could attain higher throughput with low $\gamma_{th}$ even with the presence of other devices’ interference and could then serve as a relay. If a device switches to D2D mode, the throughput of device 1 increases and so does the portion of throughput it gives to D2D devices. For the case where device 2 decides, if it serves as a relay, to use 90% of the throughput for itself and give just 10% of it to D2D devices, the mixed equilibrium is reached if device 2 communicates through D2D while device 1 serves as relay and device 3 communicates through both modes. This is because allowing a small fraction of throughput to D2D devices leads to a decrease in their throughput values.

However, choosing a mixed strategy by being indifferent doesn’t always guarantee high throughput. For example, in our case, the throughput of the devices while being indifferent is lower than the one while using pure strategies. Thus, we decide to consider the long-term behavior of the devices, by allowing them to seek the pure strategy at each part of time where the channel remains constant before it changes randomly.

### 6.3. Long-Term Behavior

We turn now to investigate the long-term behavior of the devices equilibria. The long-term behavior in our case refers to what happens to the relaying probability as the number of iterations gets big and as long as the channel is randomly changing. If the channel is experiencing a fast fading, it could not converge to a pure strategy, but it could, at each part of the time where the channel is steady, converge to the pure equilibrium that changes when the channel gain changes too. The long-term behavior is then the average relaying probability of the devices as long as the channel changes.

**Figure 11:**
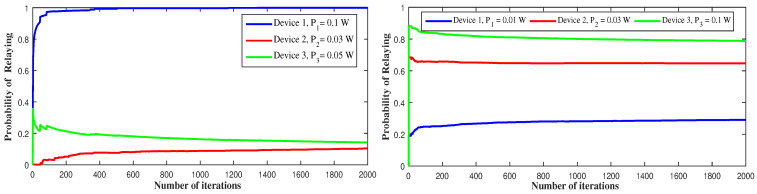
Long-term relaying probability under different transmit power schemes.

Figure 11 depicts the long-term behavior of the devices with respect to the transmit power. In the first figure, device 1 uses a higher transmit power, so it could benefit from higher throughput. This allows device 1 to use cellular mode and act as a relay for approximately all the time, while device 2 and device 3 use D2D mode for more than 80% of the time. In the second figure, device 3 uses high transmit power, so it chooses to serve as a relay for 80% of the transmission period while device 1 transmits through D2D mode for 70% of the time. This is because, when device 3 transmits with high power, it can attain high throughput values but causes high interference effect to device 1 and device 2. That is why device 1 switches to D2D for most of the time to avoid other devices’ interference and device 3 stays at the cellular side as the device offering the highest throughput.

**Figure 12:**
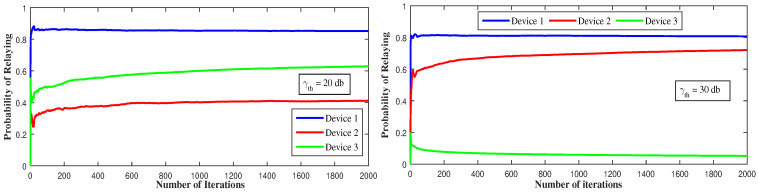
Long-term relaying probability under fast fading and different SINR threshold values.

Figure 12 represents the long-term behavior of the devices with respect to $\gamma_{th}$. Having a low SINR-threshold value allows device 1 to reach high throughput even in the presence of interference so, as the strongest device, it serves as a relay most of the time even if the channel quality changes. The same thing happens to device 2 and 3: having a low $\gamma_{th}$ allows device 3 to serve as relay more than half of the time because it could reach high throughput. Additionally, as it is the weakest device, it is not affected by other devices’ interference once on the cellular side.

**Figure 13:**
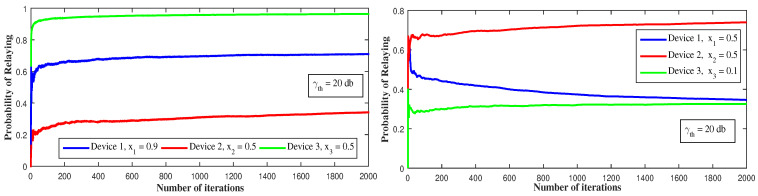
Long-term relaying probability under fast fading and different cooperation levels $x_{i}$.

Figure 13 shows the long-term behavior of the devices with respect to $x_{i}$. If device 1 decides, as a relay, to use 90% of the throughput for itself, device 3 decides then to serve as relay instead of device 1 to ensure high throughput for D2D devices. While if device 3 uses 10% of throughput for itself, its probability of relaying diminishes because its throughput decreases. Depending on the devices’ parameters, the learning algorithms allow them to converge to pure and mixed strategies to attain equilibrium. These algorithms are totally decentralized. Each device has no information about the number and the parameters of other devices. The only thing it needs is its own throughput value at each transmission.

## 7. Conclusions and Perspectives

In this article, we considered the uplink case wherein *n* devices choose whether to communicate through cellular (e.g., 5G/6G) or via D2D links to maximize their throughput. Cellular devices use NOMA, whilst they may serve neighboring devices using an orthogonal multiple access method (e.g., OFDMA/SC-FDMA). In the first part [12] of this article, we exhibited a biform game framework to predict and assess the devices behavior. In this second part, we proposed two distributed reinforcement learning algorithms to enable devices to self-explore their optimal strategies in a fully distributed manner. The simulation results showed that D2D relaying improves the overall throughput. Decentralized RL was shown to be efficient and rapid when implementing self-configuring features. However, reaching an equilibrium point does not always guarantee a satisfactory quality of service level for the devices. In our future work, we aim to deal with the case of QoS-sensitive (AR/VR/MR/XR, URLLC, etc.) applications, using federated learning.

## Figures and Tables

**Table 1:** Main symbols and their meanings.

Symbol	Meaning
*n*	Number of active devices in the cell
$P_{i}$	Transmission power vector of device *i*
$P_{i}^{c}$	Transmission power of device *i* while transmitting to the BS
$P_{i}^{d}$	Transmission power of device *i* while transmitting over the D2D link
$d_{i}$	Distance between device *i* and the BS
$h_{i}$	Channel gain of device *i*
$\gamma_{i}$	SINR of device *i*
$\gamma_{i,th}$	SINR-threshold
$P_{i}^{out}\left(\gamma_{i}\right)$	Outage probability of device *i*
$\frac{1}{\lambda }$	Mean of the channel gain
*R*	Transmission rate
$f_{j,k}$	Orthogonality factor between the carriers allocated to active devices *j* and *k*
$\alpha_{c},\alpha_{d}$	Path-loss exponent in cellular and D2D, respectively
$x_{i}$	Fraction of throughput relay *i* gives to D2D devices
$U_{i}^{t}\left(a_{i}\right)$	Utility of device *i* at time t when choosing the action $a_{i}$
